# Evaluation of the ecological health and food chain on the shores of four River Nile Islands, Egypt

**DOI:** 10.1007/s10661-022-09959-w

**Published:** 2022-03-30

**Authors:** Adel A. Abdelmageed, Radwan G. Abd Ellah, Amaal M. Abdel-Satar, Soad S. Abdel Gawad, Nehad Khalifa, Shymaa S. Zaher, Amal A. Othman, Dalia M. Belal, Howayda H. Abd El-Hady, Salem G. Salem, Mohamed H. Abdo, Amany M. Haroon, Alaa El-Far, Mahmoud H. Hegab, Engy Elhaddad, Dina M. El-Sherif, Afify D. G. Al-Afify

**Affiliations:** National Institute of Oceanography and Fisheries (NIOF), Cairo, Egypt

**Keywords:** Water quality, Biological parameters, Biochemical analysis

## Abstract

This study was conducted to evaluate the impact of human activities on the ecological health and food chain at the shores of four Nile Islands in Great Cairo including El-Qeratten, El-Waraq, El-Zamalek, and El-Manial. Water quality, bacteria, phytoplankton, benthic algae, zooplankton, macrobenthic invertebrates, and bony fishes were examined at each island shore over two seasons including the lowest and the highest flow (February and July 2020, respectively). The obtained results showed that the average values of most of the chemicals in Nile Island’s water were within the Egyptian water quality limits. Discriminant analysis of physicochemical factors revealed a similarity between El-Waraq and El-Qerateen and between El-Manial and El-Zamalek. El-Qeratten was the most polluted island. It has the highest total and fecal coliform bacteria count (3.155 and 3.050 Log MPN/100 mL, respectively). El-Zamalek shores have the highest phytoplankton (33,582 cells × 10^4^ L^−1^) and zooplankton count (310,891 organisms × m^−3^) and phyto-biochemical values. Biochemical analysis of phytoplankton demonstrated the richness of the bulk by protein (> 85% of biomass), indicating that phytoplankton has a high nutritional value. Elevated zooplankton count was recorded at El-Zamalek, which coincided with the peak of phytoplankton abundance. Mollusca were the dominant macrobenthic invertebrates on most of the island’s shores. Bony fishes were represented by 27 species and two crustaceans. The levels of the metals in fish samples were compared with the food safety guideline endorsed by the World Health Organization and Food and Agriculture Organization (WHO/FAO) to evaluate the toxicity level.

## Introduction

Freshwater resources have an important place among other natural resources. Egypt is the Nile (Burn, 2018). The Nile River is the most significant source of freshwater in Egypt. The Nile flows through the very crowded Great Cairo and support water for domestic, agriculture, and industrial activities (Al-Afify & Abdel-Satar, 2020). The impact of agricultural runoff and industrial and urban wastes increases contamination (Othman et al., 2020). Polluted water not only affects human health directly when consumed but also indirectly affects when it is used to irrigate food crops (Hussein et al., 2021).

There is a lack of routine monitoring systems in Egypt for identifying and determining the various types of contaminants in the Nile River environment, which consists of various forms of persistent organic pollutant loads (Shalaby et al., 2018). Changes in the Nile River water quality are affected not only by water management measures through the Nile barrages, but also by water and land usage for agricultural, industrial, and recreational purposes. The pollutants can enter the Nile system by direct discharges or surface runoff (Abdel-Satar et al., 2017). After the construction of the Aswan High Dam, the flow regime of the Nile has changed to a dam-regulated flow. The discharge in the summer is double that in the winter, with a water-level difference of approximately 2.5 m (Mohamed, 2019). The increase in pollution caused by the low water level in the Nile has become Egypt’s major problem, particularly since the completion of the Grand Ethiopian Renaissance Dam (Abdel-Satar et al., 2017; Heggy et al., 2021).

Burn (2018) indicated that some life cycles of the Nile food chain become more difficult when the balance of nutrients changes, especially with the number of scavenger and decomposer organisms increasing due to greater mortality rates. The abundance and biodiversity of planktons and other living organisms respond quickly to changes in the aquatic environmental variables, their abundance, and biodiversity serve as an ecological indicator of the aquatic environment (Salem & Mageed, 2021). To maintain the high water quality in a river system, the identification of fecal contamination sources is crucial (Maes et al., 2022). Plankton, macrobenthic invertebrates, macrophytes, and fishes serve as a bioindicator for the environmental status in a given time (Zakaria et al., 2016, 2018; Farrag et al., 2019).

The Nile River has hundreds of islands from Aswan to the Mediterranean Sea. In recent years, the majority of Nile River Islands have undergone rapid changes in land use (Taha, 2014). The continuing trend of urbanization has resulted in significant increases in urban areas, reducing the amount of available green spaces. Consequently, there has been a change in the morphology of the river around the islands (Raslan & Salama, 2015). Many of them have been developed in the Great Cairo region because of the construction of the Aswan High Dam. Nile Islands are economically valuable as they have significant growth potential. Changes in the hydraulic characteristics and human interventions affect the processes of erosion and sedimentation on the islands (Sadek, 2013). Nile Islands are considered the real breathing area concerning their habitats and biological diversity in addition to their global interest. They currently represent one of the most important wintering grounds for water birds in Egypt that migrate through these islands (Bunbury, 2019).

The Great Cairo area has four large permanent islands: El-Qeratten, El-Waraq, El-Zamalek, and El-Manial. These islands were occupied by human activities such as cultivation, residential development, and industrialization (Bunbury, 2019). The literature indicates a paucity of information describing the freshwater biodiversity across the Nile Islands areas. In addition, there are very few studies on the health assessment of the Nile Islands. Moreover, there are no detailed studies on the Nile Islands in Egypt regarding their shore environment and biodiversity to improve their local environment. This study was conducted to evaluate the ecological health of four River Nile Islands in Great Cairo based on the water quality and biodiversity assessment. All the Nile Islands are important and in the assessment by the program of the National Institute of Oceanography and Fisheries, especially after the Grand Ethiopian Renaissance Dam work, where all Nile shores may be affected by the decrease in the water budget due to the Nile’s altered flow which will increase the existing water deficit for Egypt (Heggy, 2021).

## Material and methods

### Study area

**Fig. 1:**
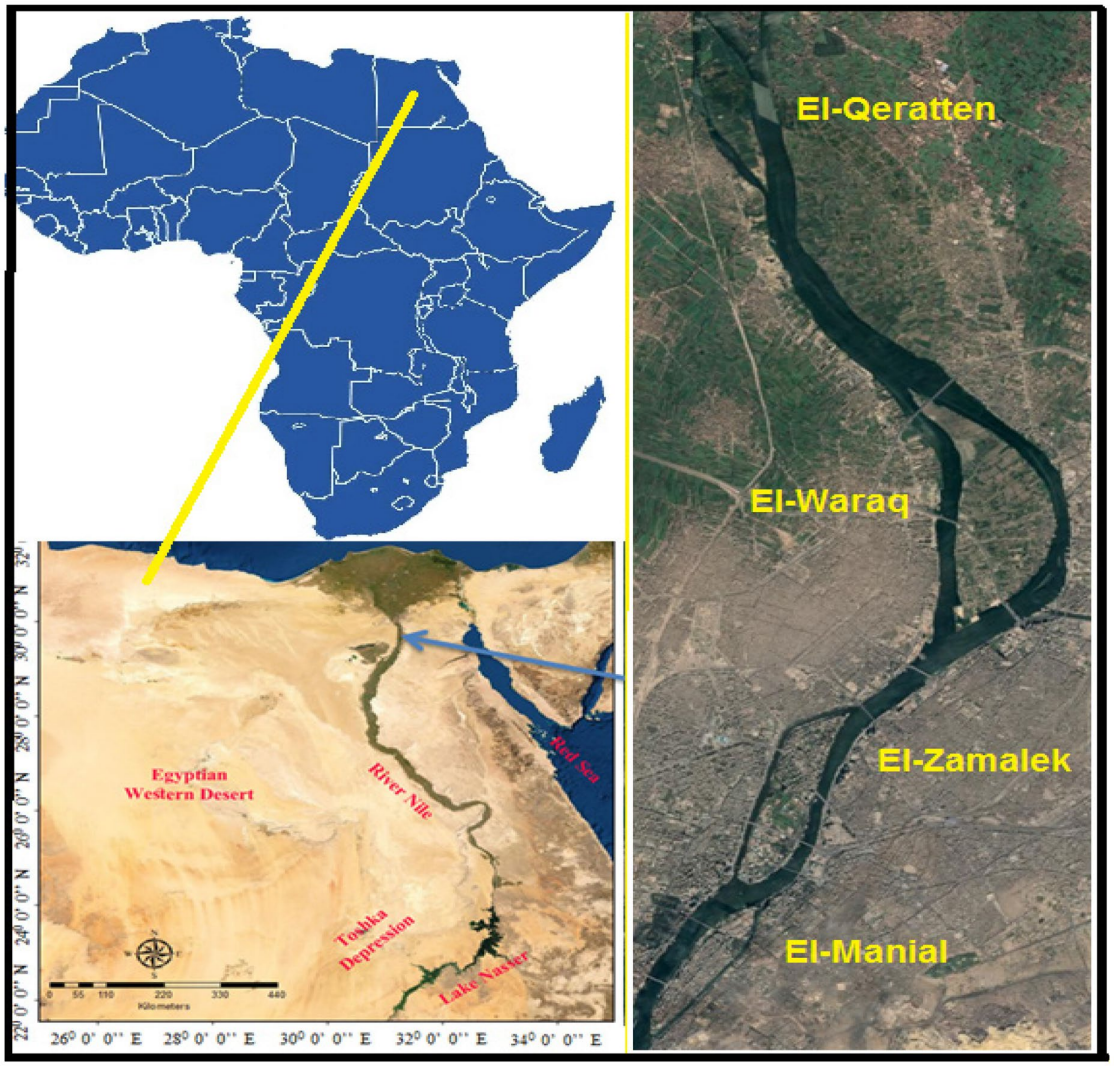
Egypt and studied four islands in the River Nile

This study is focused on four River Nile Islands with three features, namely, agricultural activities only (El-Qeratten), agricultural activities and inhabitant residences (El-Waraq), and inhabitant residences only (El-Zamalek and El-Manial), as in Table 1 and Fig. 1. The survey program of the study was implemented between February (the lowest flow season) and July 2020 (the highest flow season). Six stations were examined which are located on surrounding islands.

**Table 1:** Morphometric information of the four River Nile Islands

Island	Length (km)	Maximum width (km)	Area (km^2^)	Feature	Water depth (m)
El-Qeratten	3.8	0.9	2.6	Mostly agricultural land, with few residential areas	(< 1–10.2)
El-Waraq	5.0	1.7	7.3	Mostly residential areas and agricultural lands	(< 1–17.2)
El-Zamalek	3.9	0.9	3.6	Mostly residential areas	(< 1–13.6)
El-Manial	3.2	0.7	2.1	Mostly residential areas	(< 1–12.6)

### Morphometric features and water quality

Water depth was measured using a portable echosounder device (Lowrance Sonar-X-25 and Navman, fish 4500). Water samples were collected from the subsurface for each station using a water sampler and then transferred into well-cleaned plastic bottles. Water temperature, electrical conductivity (EC), and pH were measured in the field using Con 500 conductivity/TDS/temperature meter and combined meter pH/EC/TDS/temperature (Mi 805). Transparency was measured using a Secchi disk (25 cm). Physical and chemical analyses were conducted according to APHA (2005). Total solid (TS) content was measured by evaporating a known volume of a well-mixed sample. Dissolved oxygen (DO) content was assayed using a modified Winkler method and calculated as a percentage. Biological oxygen demand (BOD_5_) was determined using the 5-day incubation method. Chemical oxygen demand (COD) was evaluated using the potassium dichromate method. Nitrate was measured as nitrite after cadmium reduction. Total phosphorus (TP) was estimated as reactive phosphate after persulfate digestion using the ascorbic acid–molybdate method. Sediment samples were collected from the four islands using the Ekman device from the top 20 cm layer of the bottom. Grain size analysis was performed using the dry sieving technique (Folk, 1980), and the sediment organic matter (OM) was estimated using the wet oxidation method (Loring & Rantala, 1992).

### Biological methods

For microbiological analysis, water samples were aseptically collected in 200-mL sterile brown bottles, transported to the laboratory, and stored at 4 °C. Bacteriological analysis was completed within 48 h of sampling; total bacteria were enumerated on plate count agar medium at incubation temperatures of 22 °C and 37 °C. The most probable number (MPN) procedure was used to determine the total coliform (TC) and fecal coliform (FC) counts using lauryl tryptose broth (LTB) (35 °C ± 0.5 °C at 24 ± 2 h) for TC, EC broth (44.5 °C ± 0.2 °C at 24 ± 2 h) for FC, and azide dextrose broth for fecal streptococci (APHA, 2005). Total diazotrophs (TS) were enumerated using the surface-inoculated plate method on N-deficient combined carbon sources agar medium (Hanna et al., 2013). Samples for analyzing phytoplankton communities were collected using a 1.5-L Ruttner sampler, which was preserved immediately with 4% neutral formalin and then transferred into a glass cylinder, after which Lugol’s iodine solution was added (APHA, 2005). The phytoplankton count (cells L^−1^) was estimated using an inverted microscope. Epipelic diatom samples were collected using an Ekman grab sampler from the surface layer. Diatom sample preparation, diatom cleaning, and counting were conducted according to ANS (2002). For phyto-biochemical analysis, water samples were collected, sieved, and filtered through a 100-μm-sized mesh net. Then, 10 mL of the filtered water was refiltered using Whatman GF/F (0.7-μm pore diameter) fiber circles. Total protein content was determined by the Biuret method (Brown, 1984). Carbohydrate content was measured according to the phenol–sulfuric acid method (DuBois et al., 1956), and total lipid content was determined using the sulfo-phospho-vanillin procedure (Anschau et al., 2017). Elemental composition was determined as described by Vollenweider (Vollenweider, 2000). Zooplankton samples were collected from each site by filtering 30 L of surface water through a 55-µm mesh plankton net. The collected samples were preserved in plastic bottles containing 4% formalin. Zooplankton count was expressed as the number of organisms per cubic liter. Samples of macrobenthic invertebrates (MBI) were collected using an Ekman grab sampler. The collected samples were washed in a sieve of a mesh diameter of 500 μm and then preserved in a 7% neutral formalin solution. In the laboratory, the samples were washed again through a net with a mesh diameter of 0.5 mm and sorted according to genera or species, and each taxon was identified and counted. The population density was calculated and expressed as organisms per square meter. Submerged macrophytes were sampled using a grapnel to collect relative standing crop samples (five grapnel hauls per sampling area) (Ali et al., 2007). The presence of species was expressed as a percentage of sites with taxa. Fish samples were collected using commercial and experimental fishing gears and methods. Samples were maintained freshly in an icebox and transferred to the laboratory for taxonomic analysis to the species level according to Bishai and Khalil (1997) and FishBase (Froese & Pauly, 2019). For heavy metal (HM) analysis of fish muscles and gills, a small piece of tissue sample (0.5 ± 0.01 g) was digested using acid-washed digestive vessels of Teflon as described by Goldberg et al. (1983). The metal concentrations in the extracts were analyzed for total Fe, Mn, Zn, Cu, Pb, and Cd levels using GBC atomic absorption reader model Savant AA-AAS with GF 5000 graphite furnace. HM concentrations were determined on a ppm wet weight basis.

### Analytical methods

Data were statistically analyzed to evaluate the characteristics of the different islands. Discriminant analysis (DA) was conducted for physicochemical factors for the different collection sites in the four islands. Bacteria, plankton, and bottom fauna were described and related to major environmental gradients using canonical correspondence analysis (CCA) using the XLSTAT 2016 software. The Mstat program (v.4, 1982) was used for the analysis of variance to investigate significant effects at *p* < 0.05.

## Results and discussion

Aquatic food chains are affected by certain environmental contaminants. Pollution, eutrophication, and human activities are recognized as primary threats to freshwater biodiversity and river health (Abdel-Satar et al., 2017).

### Physicochemical characteristics of water and sediments

The mean and range of the physicochemical characteristics of the four islands are presented in Table 2, showing the comparison of the islands’ water variables with Egyptian water quality limits (EWQL), which were specified for freshwater according to Law 48 of 1982 regarding the protection of the Nile River from pollution. In all islands, no spatial variations (*p* > 0.1) were recorded for all the examined variables. The water temperatures were low in February compared with those in July. In contrast, the pH values, conductivity, TDS, and transparency were high in February compared with those in July (Table 2). Overall, the islands were slightly basic with comparable mean pH values. The average DO values in all the examined islands were within the water quality limits endorsed by EWQL and showed significant temporal variations (*p* < 0.001), whereas the water samples collected in July showed the lowest DO percentage, but no significant differences were obtained (*p* > 0.05) in DO values. El-Qeratten had the lowest mean value for TP and suspended solids. The mean nitrate–N concentrations were the highest in El-Zamalek. The mean COD and BOD values decreased at El-Qeratten and El-Manial, whereas the BOD/COD ratio was > 0.6. Al-Afify et al. (2018) reported that nitrate and BOD values can be considered indicators of pollution in the Nile River. Nitrate and TP levels correlated significantly (*r* = 0.60, 0.82, 0.80, and 0.65; *n* = 12, *p* < 0.01, for El-Qeratten, El-Waraq, El-Zamalek, and El-Manial, respectively), indicating the common nutrient salt sources from industrial, domestic, and agricultural wastes as the main sources of N and P inputs into the Nile River water. This leads to biodiversity changes and disturbances in benthic algal, phytoplankton, and macroinvertebrate biomass inhabiting the Nile River (Abdel-Satar et al., 2017; Othman et al., 2020). The bottom sediments of El-Qeratten and El-Waraqwere are sandy mud, whereas those of El-Zamalek and El-Manial were sandy. Sediment OM content was the highest at El-Zamalek shores, followed by El-Qeratten, whereas El-Manial had the lowest content.

**Table 2:** Mean and range values of physicochemical water determinants and sediment analysis on the islands’ shores

	El-Qeratten	El-Waraq	El-Zamalek	El-Manial	Egyptian water quality limits*
Temperature (°C)	17.7–12.1	17.4–29.8	17.4–28.5	17.4–26.9	
Transparency (cm)	80–110	90–120	85–100	80–105	
Ec (μS)	370–418	370–373	363–423	373–343	
pH	8.28 (8.59–7.58)	8.48 (8.61–8.31)	8.12 (8.49–7.64)	8.22 (8.62–7.78)	7–8.5
DO (% saturation)	96.56 (131–64.6)	102.56 (134.74–76.13)	100.34 (137.95–74.72)	106.45 (146.71–69.96)	> 61% (> 5 mg L^−1^)
COD (mg L^−1^)	7.10 (8.8–6.16)	7.52 (9.02–5.8)	7.39 (8.2–6.3)	6.90 (8.8–5.52)	< 10
BOD (mg L^−1^)	5.15 (6.30–3.38)	5.67 (6.96–4)	5.85 (6.6–4.6)	5.49 (6.4–4.6)	< 6
Nitrate-NO_3_ (µg L^−1^)	81.20 (161–18.8)	78.54 (163.9–25.3)	103.27 (256.58–10.78)	81.48 (261.85–22.40)	< 45,000
Total phosphorus (mg L^−1^)	2.14 (4.55–0.07)	2.08 (4.70–0.09)	1.14 (3.10–0.15)	1.70 (3.36–0.09)	
Total solids-TS (mg L^−1^)	275 (662–312)	265 (584–332)	256 (548–340)	276 (692–316)	< 500
Sediment texture	Sandy mud	Sandy mud	Sand	Sand	
Sediment organic matter (%)	8.9	5.1	12.5	2.5	

*Limit specified for freshwater, according to Law 48 of 1982, regarding the protection of the Nile River and waterways from pollution in Egypt

**Fig. 2:**
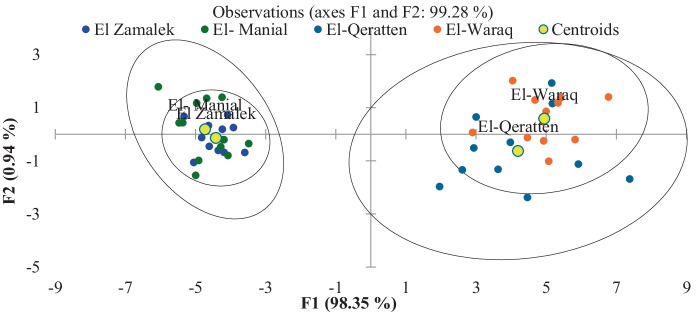
Discriminant analysis according to physicochemical variables of islands water

The results of the DA for the physicochemical variables in the four islands showed a similarity between El-Waraq and El-Qeratten and between El-Manial and El-Zamalek (Fig. 2). The first two islands were dominated by agricultural activities, while the other two had residences only.

### Biodiversity

The number of phytoplankton, attached diatoms, zooplankton, MBI, macrophytes, and fish populations found in all samples followed a consistent pattern, with the mean richness in diversity for the four islands following the order: El-Qeratten > El-Zamalek > El-Waraq > El-Manial (Table 3). El-Qeratten showed more richness in diversity than the other islands’ biota, particularly in terms of phytoplankton and attached diatoms. Evaluation of the difference between the minimum and the maximum number of recorded species as well as the overall number of species at each island revealed the following order: El-Qeratten > El-Manial > El-Zamalek > El-Manial.

**Table 3:** Total number of species recorded in the studied islands

	El-Qeratten	El-Waraq	El-Zamalek	El-Manial
Phytoplankton	90	75	81	69
Attached diatoms	58	47	43	36
Zooplankton	38	23	23	21
Bottom fauna	19	16	18	23
Fishes	24	22	22	29
Macrophytes	4	4	na	2

### Changes in food chain

#### Bacteria

As shown in Table 4, the different bacterial counts significantly varied (*p* < 0.05) depending on the study islands. The differential temperature ratio test, in which the ratios of bacteria grown at 20 °C to those grown at 35 °C were less than 10 compared with the permissible standard of 10:1 (Directive, 1998), suggested that the island’s water was heavy pollution. Although the presence of pathogenic bacteria is not necessarily related to a high FC value, a high FC value implies poor water quality and increased health risk associated with the presence of pathogenic microorganisms (Farhadinejad et al., 2014). In general, the sampling site has an impact on the bacteriological quality of the water being examined. Land use and expanded animal–agriculture systems can have an impact on FC. The principal factors influencing bacterial pollution in agricultural streams are reported to be livestock and manure application as fertilizers (Pandey et al., 2014). Most households in the rural areas, such as in El-Qeratten, lack access to sewer systems, or the usage of individual septic tanks, resulting in untreated sewage being discharged into rivers, perhaps leading to fecal contamination (Al-Badaii & Shuhaimi-Othman, 2015; Othman et al., 2020). Associative nitrogen-fixing bacteria (diazotrophs) were found in significant population densities in the islands’ water, indicating the intrusion of agricultural drainage waters into the island’s Nile water. Diazotrophs play a vital role in the aquatic system, not only as N_2_ fixers but also they increase water productivity by releasing growth factors that are beneficial for flora and fauna (Othman et al., 2016). The diazotroph bacteria that are capable of growth on N-deficient combined carbon source medium were also detected in mid-stream of River Nile water where their values ranged between 10^2^ and 10^4^ (Ali et al., 2011). El-Qeratten had unusually high average levels of TC and FC bacteria when compared to other islands, while fecal streptococci were found in the lowest count. For monitoring microbiological water quality, FC counts are used as a guideline.

**Table 4:** The average number of different bacterial groups was recorded at the four study islands

	Total coliforms	Fecal coliforms	Fecal streptococci	Total counts at 37 °C	Total counts at 22 °C	Total diazotrophs
	(Log MPN/100 ml)			(Log CFUs /ml)		
El-Qeratten	3.155^a*^	3.050^a^	0.878^c^	3.012^c^	2.703^c^	2.800^d^
El-Waraq	2.601^c^	2.401^b^	1.271^a^	3.672^a^	3.202^a^	3.684^b^
El-Zamalek	2.778^b^	2.304^c^	1.168^b^	3.672^a^	3.164^b^	3.619^c^
El-Manial	2.457^d^	2.080^d^	1.259^a^	3.355^b^	3.218^a^	3.820^a^
L.S.D. 0.05	0.018	0.069	0.026	0.026	0.018	0.018
Permissible levels		< or = 10^2wh^°, < or = 10^3wh^°		10^EEC^	100^EEC^	

(WHO, 2006), permissible levels of fecal coliforms/100 ml, unrestricted irrigation < or = 10^2^, restricted irrigation < or = 10^3^, *EEC* European Economic Community

*Mean values followed by the same letter are not significantly different

#### Phytoplankton

**Fig. 3:**
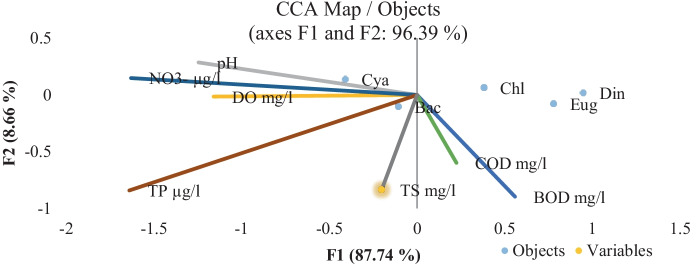
Canonical correspondence analysis (CCA). Ordination diagram of phytoplankton classes; Bacillariophyceae (Bac), Chlorophyceae (Chl), Cyanophyceae (Cya), Dianophyceae (Din), Euglenophyceae (Eug) data together with physicochemical variables: pH, DO, COD, BOD, nitrate–N, TP, and TS

Phytoplankton abundance was represented by five classes: Bacillariophyceae, Chlorophyceae, Cyanobacteria, Dinophyceae, and Euglenophyceae, in order of their abundance, except in El-Zamalek where Cyanophyceae occupied the second predominant position. Cryptophyceae and Chrysophyceae were very rare classes in the islands. El-Qeratten showed the highest diversity of phytoplankton species, where 90 species were identified. The lowest species diversity was recorded at El-Manial (69 species). Phytoplankton abundance and species composition revealed slight variation between the four islands. El-Qeratten shores water had the lowest counts (16,112 cells × 10^4^ L^−1^), while El-Zamalek shores had the highest counts (33,582 cells × 10^4^ L^−1^), Table 6, with the highest levels of nitrate and BOD (103.27 µg L^−1^ and 5.85 mg L^−1^, respectively) recorded in its water. High nutrient concentrations as a limiting factor influenced phytoplankton distribution and growth, in addition to macrophyte density (Haroon & Hussian, 2017). Phytoplankton distribution in the islands was significantly affected by environmental variables. CCA (Fig. 3) indicated that diatoms were affected by COD, BOD, and TS, whereas the presence of blue-green algae was highly affected by pH, as they tend to grow better at a high pH. Through photosynthesis and respiration, there is a relationship between algal development and oxygen levels (DO, COD, and BOD) in the aquatic ecosystem (Ma et al., 2021).

**Fig. 4:**
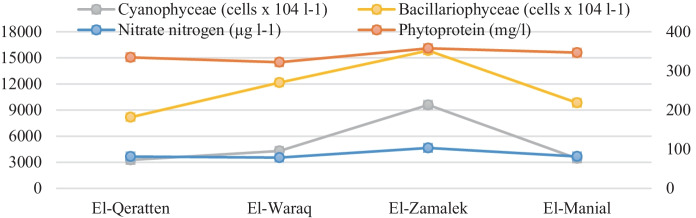
Comparison of the phytoprotein contents (mg l^−1^), NO_3_^−^-N (µg l^−1^) and the two main phytoplankton groups (Cyanophyceae and Bacillariophyceae, cells × 10^4^ l^−1^) at the four islands

Phyto-biochemical analysis revealed the richness of phytoplankton bulk by protein (> 85% of biomass), followed by carbohydrates and lipids (Table 5), indicating that phytoplankton has a high nutritional value. Protein contents indicated that phytoplankton is physiological healthy with high relative growth rates, and high protein content suggests that phytoplankton had no nitrogen depletion in the water body (Abd El-Hady et al., 2016). The highest phyto-biochemical contents were recorded at El-Zamalek (363.41 mg L^−1^). This elevation may be related to increasing nitrate levels (103.3 µg L^−1^) at the island (Fig. 4), wherein a strong correlation was observed between phytoplankton proteins and nitrate at El-Zamalek (*r* = 0.96). The N-sufficient condition can increase the cellular algal content of proteins where nitrogen represents a critical macronutrient that regulates metabolism and consequently the biochemical composition and growth of microalgae (Zarrinmehr et al., 2020). The increase in the phytoprotein contents at El-Zamalek was related to the highest phytoplankton counts (33,582 cells × 10^4^ L^−1^), with Cyanophyceae and Bacillariophyceae representing the two largest groups (9583 and 15,833 cells × 10^4^ L^−1^, respectively). Cyanophyceae have a high crude protein content (60–65%), which is the most component of cyanobacterial biomasses (Niccolai et al., 2019). Under climatic conditions, the carbon allocation pattern reveals a high proportion of proteins, which is required to support Bacillariophyceae’s rapid growth rate (Wagner et al., 2017).

**Table 5:** Biochemical contents (mg L^−1^), elemental composition (mol) measured in the phytoplankton cells from the four islands during the study

	El-Qeratten	El-Waraq	El-Zamalek	El-Manial
Phytocarbohydrates	4.9	5.1	4.98	4.58
Phytoproteins	334.43	322.17	357.63	346.6
Phytolipids	0.81	0.72	0.8	0.72
C	179.63	173.25	192.02	185.94
N	57.86	55.74	61.87	59.96
H	23.8	22.95	25.42	24.66
O	78.76	76.05	84.09	81.37

Elemental ratios (C, N, H, and O) of phytoplankton provide insight into the mechanism by which nutrients in the water body are allocated within algal cells to support cellular functions, growth, and metabolism. The carbon contents of phytoplankton in the four investigated islands were > 50% of elemental compositions. The high incorporation of carbon into the phytoplankton biochemical structure was a characteristic pattern of the photosynthetic allocations of healthy phytoplankton biomass (Schulhof et al., 2019). The ideal C/N ratio, according to Redfield et al. (1963), is 6.6. However, phytoplankton progressed quickly, with the C/N ratio dropping to 3.1 along the study island’s shores with the increase in phytoprotein, indicating that the algae were not nitrogen-limited (Napiórkowska-Krzebietke et al., 2015). Protein represented the largest pool of cellular C and N in algal species under steady-state growth (Liefer et al., 2019; Islam et al., 2019).

#### Epipelic diatoms

**Fig. 5:**
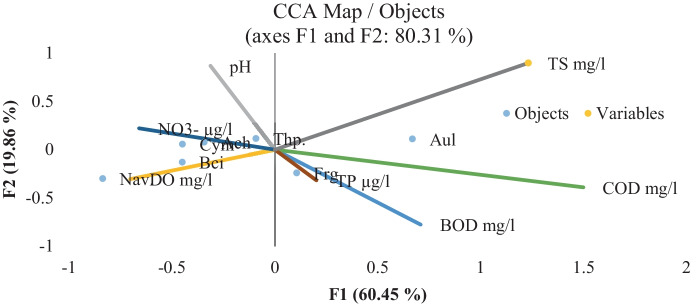
Canonical correspondence analysis (CCA). Ordination diagram of the attached diatoms orders; Achnanthales (Ach.), Aulacoseirales (Aul.), Bacillariales (Bci.), Cymbellales (Cym.), Eunotiales (Eun.), Fragilariales (Frg.), Naviculales (Nav.), Rhopalodiales (Rh.), Thalassiophysales (Thp.), and Thalassiosirales (Ths.) data together with physicochemical variables: pH, DO, COD, BOD, NO_3_^−^-N, TP, and TS

The attached epipelic diatoms were represented by 10 orders with a total of 80 species. The most dominant order (> 50% of the total count) was Thalassiosirales, which had roughly comparable counts on all four islands, showing great cosmopolitanism (Table 6). El-Qeratten shores had the highest number of species, followed by El-Waraq, which is consistent with the high TP concentration. According to CCA (Fig. 5), diatoms highly correlated with TP and the sandy mud sediment texture. The species composition of benthic diatoms responds directly to nutrients, with total N or P concentrations increasing up to a point before leveling off. The region above which primary producers have not developed to adapt to nutrient concentration could be the point where the autotrophic state no longer varies. CCA findings revealed that the studied environmental variables can explain 40% of the diatom community variation. Fragilariales was substantially influenced and correlated with TP, whereas Thalassiosirales was near the zero point, demonstrating a cumulative effect of the different environmental variables. Fragilariales were highly affected and correlated with TP. The response of the same species to similar nutrient enrichment conditions can be different, suggesting that factors other than nutrients have a combined effect with the nutrient in determining species composition and other physical factors (Yang et al., 2015). According to the DA, the diatom data can characterize or separate El-Waraq and El-Manial in only a few points, whereas most points had the same pattern and could not separate the islands into distinct clusters. This shows that there are no major differences in the environment of the four examined islands when it comes to the community of attached diatoms. This could be due to some tolerant species cosmopolitan and the influence of other environmental conditions.

**Table 6:** The average number of the different biota recorded at the four studied islands

	El-Qeratten	El-Waraq	El-Zamalek	El-Manial
Phytoplankton classes (no. of cells × 10^4^ l^−1^):				
Bacillariophyceae	8175	12,150	15,833	9833
Chlorophyceae	4183	9308	7508	7558
Cyanophyceae	3271	4300	9583	3433
Dianophyceae	250	392	433	500
Euglenophyceae	233	400	225	233
Total	16,112	26,550	33,582	21,557
Attached diatoms orders (no. of cells × 10^4^ L^−1^)				
Achnanthales	514	247	549	508
Aulacoseirales	734	2338	703	380
Bacillariales	108	91	105	215
Cymbellales	265	129	137	328
Eunotiales	65	91	37	11
Fragilariales	2197	2682	3308	2866
Naviculales	96	125	129	628
Rhopalodiales	2	0	0	8
Thalassiophysales	28	16	10	41
Thalassiosirales	5928	4353	5022	4920
Total	9,937	10,072	10,000	9,905
Zooplankton groups (no. of organisms m^−3^)				
Rotifera	206,798	279,871	307,391	223,732
Cladocera	5667	3055	2833	2916
Copepoda	3458	3250	667	1472
Total	215,923	286,176	310,891	228,120
Bottom fauna phyla (no. of organisms m^−2^)				
Annelida	1001	1962	203	217
Arthropoda	235	573	361	517
Mollusca	1478	1161	739	1358
Total	2,714	3,696	1,303	2,092

#### Zooplankton

**Fig. 6:**
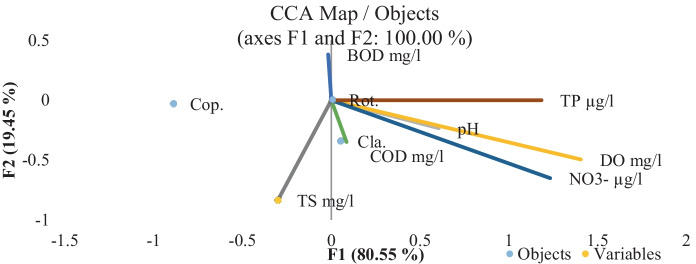
Canonical correlation analysis (CCA). Ordination diagram of zooplankton groups; Rotifera (Rot.), Copepoda (Cop.), and Cladocera (Cla.) data together with physicochemical variables: pH, DO, COD, BOD, nitrate–N, TP, and TS

Zooplankton organisms were represented by small rotifers (> 90% of total counts), copepods, and cladocerans. According to Sun et al. (2021) increased predation is critical for small-bodied zooplankton dominance in the aquatic system. The study islands’ major rotifer species are eutrophic indicator species (Palmer & Herat, 2021). As a result, the considerable dominance of rotifers over other zooplankton groups in the studied area, both in terms of abundance and species composition, provides evidence of the trophic status of the Nile River islands. Phytoplankton abundance has been reliably associated with zooplankton abundance, where the highest zooplankton count was recorded at El-Zamalek (310,891 organisms m^−3^), which coincided with the peak of phytoplankton abundance, Table 6. The variation in phytoplankton and zooplankton composition was consistent with the regulation of nutrients and top-down effects by fish (Makarewicz & Jones, 1990). Planktivore abundance increases with phytoplankton and zooplankton abundance, and these organisms selectively feed on larger zooplankton (Iglesias et al., 2011). CCA ordination of the zooplankton assemblage data (Fig. 6) demonstrated a broad continuum with TP, nitrate–N, and DO in the four islands, where El-Manial shores zooplankton assemblages were relatively distinct.

#### Macrobenthic invertebrates

**Fig. 7:**
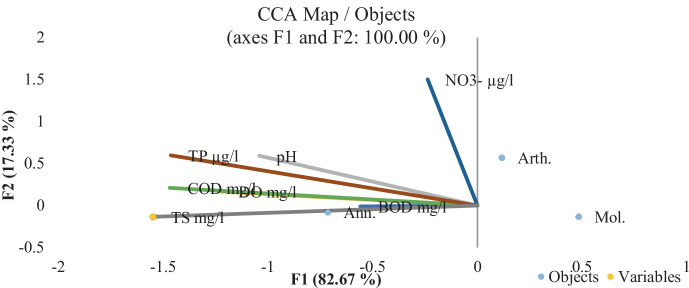
Canonical correlation analysis (CCA). Ordination diagram of bottom fauna phyla; Arthropoda (Arth.), Mollusca (Mol.), and Annelida (Ann.) data together with physicochemical variables: pH, DO, COD, BOD, nitrate–N, TP, and TS

A total of 34 species of MBI belonging to three major phyla, Arthropoda, Annelida, and Mollusca, were identified. Of these, there were 9 species of arthropods, 6 species of annelids, and 19 species of mollusks. Mollusca was the dominant group at all islands, except El-Waraq shores, where Annelida was the most dominant, indicating that the type of sediments at the islands has a specific role, where El-Waraq shores had the highest percentages of mud deposits, and Annelida preferred this sort of sediment (Abdel Gawad, 2019). El-Qeratten had the highest percentage of Mollusca, with 1478 organisms m^−2^, while El-Zamalek had the lowest number, with 739 organisms^−2^. When compared to El-Qeratten and El-Waraq, the overall number of Annelida species fell at El-Zamalek and El-Manial shores. Annelida showed positive correlations with pH and COD (*r* = 0.96 and 0.58, respectively) and negative correlations with DO and depth (*r* =  − 0.35 and − 0.63, respectively). Arthropoda, especially the larvae of Chironomidae, showed significant positive correlations with BOD and DO (*r* = 0.96 and 0.6, respectively). Mollusca exhibited considerable positive correlations with transparency and Ca^++^ (*r* = 0.75 and 0.66, respectively). The results of CCA of MBI group data (Fig. 7) revealed a continuum between the distribution of MBI in the islands with TP, TS, COD, and DO, where the distribution of each species depends on the levels and types of such chemical variables.

#### Macrophytes

Five macrophyte species were recorded, demonstrating poor species diversity in the shores of the studied islands, especially at El-Zamalek and El-Manial. They were classified into three submerged and two floating macrophyte species belonging to five families and five genera (Table 7). Among these, dense populations of the submerged macrophyte species *Myriophyllum spicatum* were recorded at 83.33% of the total sampling sites, followed by those of *Ceratophyllum demersum* (41.66%). However, the other three species were very rare. Madsen (1998) considered *M. spicatum* as an invasive species, indicating the effect of environmental factors. In this study, it was found to have a prominent effect on the community composition of submerged macrophytes, as it competes aggressively to displace and reduce the diversity of the originally present aquatic plants. Similar findings were reported by Haroon and Hussian (2017) who reported the shading effect as well as the allelopathic substances released by *M. spicatum* in reducing the growth of other submerged macrophyte species. Furthermore, El-Ghani et al. (2011) indicated that high nitrate availability and sediment OM lead to increased invasion of the submerged macrophyte species *M. spicatum*.

**Table 7:** Presence (1), absence (0), and total presence percentage (TP%) of the recorded macrophytes at the four islands

Species	Family	Q	W	Z	M	TP%
Submerged macrophytes						
*Myriophyllum spicatum* L	Haloragaceae	1	1	0	1	83.33
*Potamogeton perfoliatus* L	Potamogetonaceae	0	1	0	0	8.33
*Ceratophyllum demersum* L	Ceratophyllaceae	1	1	0	0	41.66
Floating macrophytes						
*Potamogeton nodosus Poir*	Potamogetonaceae	1	1	1	1	8.33
*Eichhornia crassipes*	Pontederiaceae	1	0	0	0	7.69

Q, El-Qeratten Island; W, El-Waraq Island; Z, El-Zamalek Island; and R, El-Manial Island

#### Fish community

A total of 29 fish species (27 bony fish and 2 crustaceans) belonging to 17 families were recorded at the investigated islands (Table 8). The occurrence percentage showed that Cichlidae was the most dominant and diversified family (97.2%), followed by the families Clariidae (95.8%), Bagridae (93.8%), Poeciliidae (83.3%), Claroteidae (72.9), and Mormyridae (70.8%), and the other families constituted < 60% (Table 9). Elsaied et al. (2021) concluded that most inland African freshwater fishes belong to two broad phylogenetic orders, Cichliformes and Cyprinodontiformes, both significantly diversify in terms of fisheries and aquaculture. The family Cichlidae is the most prosperous, omnivorous, and their feeding habits slightly vary with fish size, sex, and season (Shalloof et al., 2020; Temesgen et al., 2022). The two crustaceans included *Procambarus clarkii* and *Mediapotamon* spp*.* El-Manial had the most abundant fish species.

**Table 8:** List of fish families and species recorded in the present study

Family	Species
Cichlidae	*Coptodon zillii*
	*Haplochromis bloyeti*
	*Hemichromis bimaculatus*
	*Oreochromis aureus*
	*O. niloticus*
	*Sarotherdon galilaeus*
Cyprinidae	*Barbus bynni*
	*Cyprinus carpio*
	*Labeo horie*
	*L. niloticus*
Bagridae	*Bagrus bajad*
	*B. docmak*
Claroteidae	*Chrysichthys auratus*
	*C. rueppelli*
Mochokidae	*Synodontis clarias*
	*S. schall*
Mormyridae	*Mormyrus chachieve*
	*M. kannume*
Alestiidae	*Brycinus nurse*
Anguillidae	*Anguilla anguilla*
Atherinidae	*Atherina boyeri*
Cambaridae	*Procambarus clarkii*
Centropomidae	*Lates niloticus*
Malapteruridae	*Malapterurus electricus*
Poeciliidae	*Gambusia affinis*
Potamidae	*Mediapotamon* spp*.*
Schilbeidae	*Schilbe niloticus*
Tetraodontidae	*Tetraodon lineatus*

**Table 9:** List of fish families recorded with the percentage of occurrence in the investigated islands in the River Nile of Egypt during the present study

Family	Sp. No	Q	W	Z	M	Total
Cichidae	6	94.4	94.4	100.0	100.0	97.2
Clariidae	1	83.3	100.0	100.0	100.0	95.8
Bagridae	2	100.0	100.0	83.3	91.7	93.8
Poeciliidae	1	83.3	83.3	83.3	83.3	83.3
Claroteidae	2	66.7	66.7	100.0	58.3	72.9
Mormyridae	2	50.0	33.3	100.0	100.0	70.8
Atherinidae	1	50.0	66.7	50.0	66.7	58.3
Centropomidae	1	50.0	33.3	100.0	33.3	54.2
Cyprinidae	4	29.2	38.9	100.0	45.8	53.5
Cambaridae (crayfish)	1	54.0	50.0	49.0	47.0	50.0
Malapteruridae	1	50.0	33.3	33.3	83.3	50.0
Alestiidae	1	0.0	16.7	16.7	100.0	33.3
Anguillidae	1	50.0	16.7	16.7	16.7	25.0
Tetraodontidae	1	16.7	33.3	16.7	16.7	20.8
Potamidae (crab)	1	0.0	0.0	0.0	33.3	8.3
Mochokidae	2	0.0	0.0	0.0	25.0	6.3
Schilbeidae	1	0.0	0.0	0.0	16.7	4.2

Q, El-Qeratten Island; W, El-Waraq Island; Z, El-Zamalek Island; and R, El-Manial Island

### Oreochromis niloticus as a bioindicator of pollution

Fish are at the top of the food chain and are one of the most significant biomonitors for heavy metal pollution assessment in the aquatic ecosystem. They can accumulate metals that are passed on to humans through fish consumption, resulting in acute or chronic diseases (Yacoub et al., 2021). *O. niloticus* is the main economic fish in the Nile, used as a bioindicator of water pollution. Fe, Mn, Cu, and Zn are essential metals but can be lethal at high concentrations, whereas Pb and Cd can be harmful to living organisms and the environment at low concentrations. The levels of the metals were compared with the food safety guideline endorsed by the World Health Organization/Food and Agriculture Organization (WHO/FAO) to evaluate the toxicity level. The bioaccumulation of heavy metals was lower in muscles than in the gills. The adsorption of metals onto the surface of gills, as the first target for pollutants in water, could also be an important influence on the total metal levels of the gill. The results indicated that Fe was the most accumulated element, followed by Zn and Cu, while Cd was the least accumulated one (Table 10). The rise in Fe accumulation in fish was higher than that of other metals, possibly because of the increase of total dissolved Fe levels in aquatic media (Abdel-Khalek et al., 2020). The concentrations of Cu, Pb, and Cd in both muscles and gills, and the Zn concentration in muscles were below the WHO/FAO FSG (FAO/WHO, 2010). The concentrations of heavy metals in fish depend on the feeding habit, size, age, lifestyle, and exposure duration to contaminants (Bastami et al., 2015).

**Table 10:** Bioaccumulated metals (mg kg^−1^ weight wt) in muscles of O. niloticus, (mean size 17.5 ± 2.56 cm and mean weight 165 ± 54.45 g)

Heavy metals	Muscles	Gills	*FSG (FAO/WHO, 2010)
Fe	130.67 ± 34.78	211 ± 23.45	30.0
Zn	18.56 ± 2.23	74 ± 6.45	30.0
Mn	1.45 ± 0.45	18 ± 4.89	1.0
Cu	4.56 ± 0.12	6.45 ± 1.03	30.0
Pb	0.83 ± 0.13	1.1 ± 0.23	2.0
Cd	0.25 ± 0.07	0.45 ± 0.1	0.5

*FSG Food safety guidelines

## Conclusion

Survey monitoring program was implemented during the lowest Nile flow season and the highest Nile flow season to maintain ecological health and food chain around some Nile islands. Discriminant analysis of physicochemical factors showed high similarity between El-Waraq and El-Qeratten and between El-Manial and El-Zamalek. High average levels of TC and FC bacteria were observed at El-Qeratten compared with those at other islands, whereas the highest phytoplankton counts and zooplankton were recorded at El-Zamalek. The highest number of species of the attached diatoms was recorded at El-Qeratten. The bioaccumulation of heavy metals was lower in the muscles of *O. niloticus* than that in the gills. El-Qeratten Island was the most polluted island in comparison with other studied islands. Regular evaluation surveys and further studies should be conducted to monitor the expected changes in water sources near highly populated areas, such as the Great Cairo.

## Data Availability

Data sharing is not applicable to this article as no datasets were generated or analyzed during the current study.
